# Crack Propagation Calculations for Optical Fibers under Static Bending and Tensile Loads Using Continuum Damage Mechanics

**DOI:** 10.3390/s17112633

**Published:** 2017-11-15

**Authors:** Yunxia Chen, Yuxuan Cui, Wenjun Gong

**Affiliations:** 1 School of Reliability and System Engineering, Beihang University, Weimin Building, No. 37, Xueyuan Road, Haidian District, Beijing 100191, China; chenyunxia@buaa.edu.cn (Y.C.); cuiyuxuan@buaa.edu.cn (Y.C.); 2 Science and Technology on Reliability and Environmental Engineering Laboratory, Beijing 100191, China

**Keywords:** crack propagation, optical fiber, continuum damage mechanics, static fatigue

## Abstract

Static fatigue behavior is the main failure mode of optical fibers applied in sensors. In this paper, a computational framework based on continuum damage mechanics (CDM) is presented to calculate the crack propagation process and failure time of optical fibers subjected to static bending and tensile loads. For this purpose, the static fatigue crack propagation in the glass core of the optical fiber is studied. Combining a finite element method (FEM), we use the continuum damage mechanics for the glass core to calculate the crack propagation path and corresponding failure time. In addition, three factors including bending radius, tensile force and optical fiber diameter are investigated to find their impacts on the crack propagation process and failure time of the optical fiber under concerned situations. Finally, experiments are conducted and the results verify the correctness of the simulation calculation. It is believed that the proposed method could give a straightforward description of the crack propagation path in the inner glass core. Additionally, the predicted crack propagation time of the optical fiber with different factors can provide effective suggestions for improving the long-term usage of optical fibers.

## 1. Introduction

Optical fibers are widely investigated and applied in both telecommunication and sensor industries due to their prominent properties. On account of high capacity and low transmission loss, optical fibers are mostly used in long-haul communication systems. Additionally, they are also widely applied in sensors [1,2,3], considering their excellent flexibility and compact size. Owing to the severe environmental conditions, the optical fibers in sensors face some challenges in terms of the reliable functional guarantee and long lifetime requirement. Harsh environmental conditions could cause mechanical damage and the degradation of strength [4]. This could make optical fibers unable to satisfy the operational requirements, causing the sensors to stop working. This could bring devastating failures and costly repairs for some inaccessible devices like the micro fiber optic gyroscope (MFOG) [5], the submarine hydrophone [6] and so on. Therefore, lifetime design and corresponding improvements are essential for optical fibers to satisfy the long-term life requirement under critical operating conditions. The key issue is how to make an accurate life prediction for the optical fibers in sensors.

**Figure 1:**
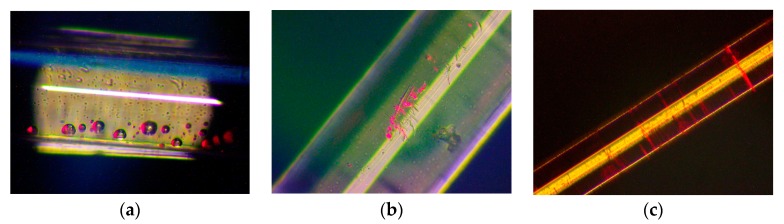
Flaws observed in the optical fiber structure: (**a**) bubbles in the polymer coating; (**b**) bruises on the fiber surface; (**c**) cracks on the fiber surface.

The common mechanical failure mode for optical fibers is the slow crack propagation at the initial stage and unsteady fast fracture. This can be determined as the static fatigue. The static fatigue-induced damage process mainly is related to the following three factors. Firstly, according to our observation shown in Figure 1, different defects including bubbles, cracks and bruises exist in the structure of optical fibers, and among these, the cracks on the surface cause the most severe damage. The fiber surface could be damaged during manufacturing processes such as post-processing or the handing step. Secondly, external tensile and bending loads place a non-uniform stress distribution on the fiber surface, which further causes the flaws to gradually grow during the operational period [7]. Furthermore, environmental effects like humidity and temperature may accelerate the damage process. Currently, the research in the field of optical fiber failure time prediction mainly includes two considerations:

- Statistical method based on the failure data.
- Mechanical method based on the crack growth model.

The statistical method needs enough failure data to support the life prediction modelling of the optical fiber. For example, Annovazzi-Ledi et al. [7] carried out static fatigue experiments with over 300 samples under different bending radii during a period of 468 days. Their statistical analysis using the maximum likelihood and the least-squares method gave an available bending radius for the lifetime requirement. In the static bending experiments of [8], more than 200 samples were used to calculate the initial strength distribution of fibers and check on the reliability measurement for optical fibers under static bending situations. Scanning electron microscope (SEM) observations conducted on tested samples exhibited the typical brittle fracture morphology, and the wedge-shape fracture always initiated from the outer silica surface with the maximum tensile stress. However, in such above experiments, environmental effects that would accelerate the failure process were ignored [9]. To face this issue, several experiments considering environmental factors such as humidity, temperature and so on were conducted. The effect of water content on the crack propagation of optical fibers has been thoroughly tested [10,11,12,13] and it was found that water can corrode the glass interface, promoting surface crack defects. It will also further accelerate the structural relaxation phenomenon. Furthermore, Armstrong et al. [12] found that the simple exponential model gives a better description than the power law of the degradation process of optical fibers under humidity. Moreover, time-to-failure prediction models of fibers were developed based on experimental results under different temperature levels, and a new method was proposed to enhance the thermal durability of optical fibers [14]. In summary, the statistical methods strongly rely on the experimental results. The test investigations just focus on the qualitative analysis of surface topography, rather than the quantitative description of the optical fiber’s failure process. Meanwhile, the mechanical method based on the crack growth model could provide a quantitative evaluation by characterizing the crack propagation process of optical fibers subjected to the static fatigue under external loads.

Currently, several empirical and mechanism-based models have been proposed for the life prediction of optical fiber. Under the static load, the fiber usually fractures from the surface of the inner glass core [8]. Hence, the glass crack growth is mainly studied. The commonly used methods are based on the stress intensity factor dominated power law. Griffioen et al. [15] reviewed and compared twelve power law-based failure-time predicting models applied on different types of fibers. They found that the applicability of the power law model to the silica fiber is questionable since it is not based on firm physical grounds. Similarly, Matthewson [16] investigated the degradation models of optical fibers, and came to the conclusion that the power law kinetic is unduly optimistic and lacks physical fundamentals. Therefore, the use of the power law is not recommended for some applications. Further study is required to yield better understanding of the crack growth mechanism in optical fibers. Therefore, various kinetics models have been proposed to describe the crack growth process in silica fibers. Lawn [17] described the crack propagation with a second exponential form model derived from the atomistic model. Matthewson et al. [18] analyzed the differences between three common models including the crack growth kinetics regression models [19,20], the model based on the simple chemical kinetics theory [21] and the atomistic crack growth model [17]. Moreover, fibers with different defects like subthreshold, post-threshold and macroscopic cracks behave quite dissimilarly and they are not expected to be described with a single subcritical crack growth model on account of residual stress. Consequently, the crack propagation subjected to combined residual and external loads is supposed to be depicted with theoretical models. In summary, the mentioned studies indicate that it is crucial to ascertain the relevant mechanism of the optical fiber fracture process to give a more accurate failure-time prediction.

In order to model the actual fracture behavior of glass with initial defects, the framework of continuum damage mechanics (CDM) has been thoroughly researched. CDM characterizes the effect of micro defects on the macro-scale properties of materials. The initial concept of CDM was introduced by Kachanov [22], then developed by Lemaitre [23] and successfully applied on quasi-brittle materials including concrete, rocks and glass. Sun [24] chose an anisotropic damage tensor to quantify the damage and cracking mechanism of glass under the static indentation situation, and a finite element simulation was generated to verify the model. Ismail et al. [25] presented a numerical simulation in which an approach of CDM combined with the fracture mechanism was performed to predict the directions of crack propagation. In the present paper, we make the assumption that a crack defect is introduced on the optical fiber coating surface during post-processing to study the crack propagation procedure and the corresponding failure time of optical fibers. A static analysis of a glass core is performed with the three-dimensional finite element model using ANSYS Workbench static analysis. A time-dependent continuum damage model for the glass core of the fiber is proposed in this study. The main content is to calculate the damage degree of each element around the crack tip, and then to simulate the crack propagation process of the glass core. Beyond that, effects of three factors including bending radius, external tensile load and optical-fiber diameter on the crack propagation path and failure time of the optical fiber are analyzed to give an optimal strategy for optical fiber applications.

The remainder is organized as follows: Section 2 introduces the optical fiber bending model and the calculation procedure of glass core crack propagation. In Section 3, the sensitivity analysis in terms of bending radius, tensile force and optical fiber diameter on the crack propagation path and failure-time prediction are presented. Additionally, experiments for verifying the finite element results are provided in Section 4. The results show that our method is suitable for the crack propagation prediction of optical fibers under static bending and tensile loads. Finally, Section 5 summarizes some conclusions.

## 2. Mathematical Modeling

### 2.1. Static Fatigue Damage Model

Based on the behavior of glass at room temperature [26,27], the constitutive equation can be formulated as follows,

(1) $$\dot{\varepsilon }={\dot{\varepsilon }}^{e}+{\dot{\varepsilon }}^{p},$$

(2) $$\varepsilon^{e}=\frac{1+\nu }{E}\tilde{\sigma }-\frac{\nu }{E}tr(\tilde{\sigma })I,$$

(3) $${\dot{\varepsilon }}^{p}=\dot{\lambda }\frac{3}{2}\frac{{\tilde{\sigma }}^{\prime }}{\sigma_{s}}.$$

The total strain rate of glass $\dot{\varepsilon }$ in Equation (1) is composed of the elastic strain rate ${\dot{\varepsilon }}^{e}$ and the plastic strain rate ${\dot{\varepsilon }}^{p}$. In Equation (2), $\varepsilon^{e}$ is the elastic strain, $\tilde{\sigma }$ is the second-order stress tensor with damage, ν is the Poisson’s ratio and *E* is the elasticity modulus. In Equation (3), $\dot{\lambda }$ is the changing rate of the instantaneous nonnegative proportionality constant, $\sigma_{s}$ is the yield stress and ${\tilde{\sigma }}^{\prime }$ is the second-order stress partial tensor. Under the constant load, glass gradually splits in optical fibers until it is totally fractured. The static fatigue mechanism of glass plays a crucial role in the failure process of optical fibers. A damage criterion is demanded to predict the static fatigue process and failure time. Since the change rate of strain in the glass is fairly tiny and could barely be acquired through the finite element analysis, a time-dependent isotropic damage revolution law for glass is introduced based on the work of Kachanov [22] and Lemaitre [28]. Assume that *D* is the accumulated damage, the damage rate $\dot{D}$ equals

(4) $$\dot{D}=\frac{\sigma_{M}^{2}R_{\nu }}{2Es{\left(1-D\right)}^{2+\alpha_{0}}}{\left[\frac{\sigma_{M}}{K\left(1-D\right)}\right]}^{n},$$

where *K*, *n* and *s* are material parameters, and $\sigma_{M}$ is the von Mises equivalent stress. $R_{\nu }$ is the triaxiality coefficient of the multiaxial state of stress that satisfies

(5) $$R_{\nu }=\frac{2}{3}(1+\nu )+3(1-2\nu )(\frac{\sigma_{H}}{\sigma_{M}}),$$

where $\sigma_{H}$ is the hydrostatic stress. The material parameters can be estimated from the relationship between crack propagation time $t_{c}$ and applied static stress in the static fatigue test of glass fibers [29]. Under the experimental condition, $\alpha_{0}=0$, $R_{\nu }=1$ and $\sigma_{M}=\sigma$. The other material parameters can be simplified as $A^{n+2}=2EsK^{n}$. The breakup time and applied stress are nonlinearly fitted by Equation (4) to obtain the values of *A* and *n*.

The crack-opening mode is assumed to be dominated by the normal tensile principle stress component above a certain damage threshold [30]. Therefore, the time-dependent damage variable can be defined as

(6) $$D=\{\begin{array}{c} 0\\ 1-{\left[1-\left(3+n\right){\left(\frac{\sigma_{1}}{A}\right)}^{2+n}\left(t-t_{\omega }\right)\right]}^{\frac{1}{3+n}}\\ 1 \end{array}\begin{array}{c} \begin{array}{l} if\;\sigma_{1}\leq \sigma_{t} \end{array}\\ \;if\;\sigma_{t}＜\sigma_{1}＜\sigma_{c}\\ \begin{array}{l} if\;\sigma_{1}\geq \sigma_{c} \end{array} \end{array}.$$

Under the static loading condition, $\sigma_{1}$ is defined as the maximum principal stress. $\sigma_{t}$ is set to be 200 MPa based on experimental results [29], which means that no damage occurs under the stress below $\sigma_{t}$. $\sigma_{c}$ is 524MPa when elements are totally damaged [31].

In addition, the following assumptions are considered:

- The material and damage variables are isotropic.
- $t_{\omega }$ is equal to 0, after which dissipation with damage occurs.
- The initial state $D_{0}$ is 0.

The damage variable *D* is calculated under the averaged stress for each element. By using the element death technique [32], elements of glass are set to inactive properties once their damage variable *D* is accumulated such that *D* = 1, and the dead elements do not contribute to the stiffness anymore.

### 2.2. Finite Element Modelling

#### 2.2.1. Description of the Finite Element Model

**Figure 2:**
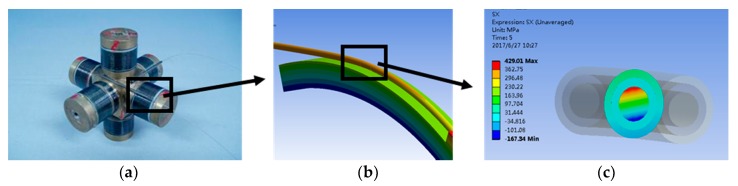
Twined optical fibers on the bobbin and partial views: (**a**) the optical fiber wound on the spool; (**b**) the partial view of the optical fiber subsection; (**c**) the cross-sectional stress of the optical fiber subsection.

In this work, the finite element model is generated in ANSYS Workbench to obtain the mechanics parameters, including strain and stress, that will be used in the calculation of damage of interested elements. Here, we use the three-dimensional optical fiber winding model in hydrophone [33] with a change of the geometry parameters and a simplification of the twining mode. As shown in Figure 2, spiral optical fibers wound on the spool are simplified as a length of fiber circumferentially twined on a part of the spool. Then, concentration is focused on the part of the optical fiber with a defect on the coating.

The optical fiber is composed of several layers. The doped glass with a higher refractive index is used to transfer the optical signal. The cladding layer is doped glass with a lower refractive index, to make sure that light is restricted to the core. The mechanical properties of core and cladding glasses are quite similar, and these two parts could be simplified as the glass core with 125 μm diameter. The glass core is coated with two protecting polymer layers whose diameters are 187 μm and 245 μm. The Young’s modulus of the inner coating is 3.5 MPa, and that of the outer coating is 6.24 GPa. The other geometry parameters as well as the material properties of the optical fiber in the simulation are listed in [33].

**Figure 3:**
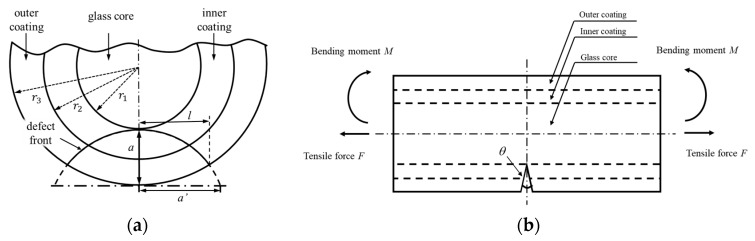
Crack defect position and optical fiber structure; (**a**) crack position and geometry on the optical fiber surface; (**b**) crack position and the external load.

On the basis of the experimental results, the optical fiber under bending circumstances fractures from the surface of the glass core. Once the glass core is broken, the light transmission of the optical fiber is stopped. Thus, the crack propagation in the glass core is focused. A penny-shaped crack is introduced on the optical fiber surface as shown in Figure 3a, which crosses the two acrylate layers and reaches the surface of the glass core. The original size of the crack is *l* = 73 μm, *a* = 60 μm. Figure 3b exhibits the crack position and the geometry on the fiber surface; the crack opening angle θ is 2 degrees.

#### 2.2.2. Calculation of the External Loads

Tensile forces are applied to the distal end section of the optical fiber, and the spool rotates around its axis for 45 degrees, leading the optical fiber to unwind on the spool surface. The stress on the cross section of the middle part is obtained to simulate the bending deformation of a part of optical fiber.

Transverse loads are applied, including tensile forces and bending moments, which are defined as

(7) $$M=M_{glasscore}+M_{coating},$$

(8) $$M_{glasscore}=\frac{E_{i}\cdot \pi \cdot d_{i}{}^{4}}{64\cdot (R+r_{i})}\;(i=1),$$

(9) $$M_{coating}=\frac{E_{i}\cdot \pi \cdot d_{i}^{4}}{64\cdot (R+r_{i})}\left[1-{\left(\frac{d_{i}}{d_{i-1}}\right)}^{4}\right]\;(i=2,3),$$

where *E_i_* is the Young’s modulus of the *i*th component layer, *d_i_* and *r_i_* are respectively the diameter and radius of the *i*th layer in the optical fiber (note that *i* = 1, 2, 3 separately represent the glass core, inner coating and outer coating layers, respectively), and *R* is the bending radius.

#### 2.2.3. Calculation of Crack Propagation Time

**Figure 4:**
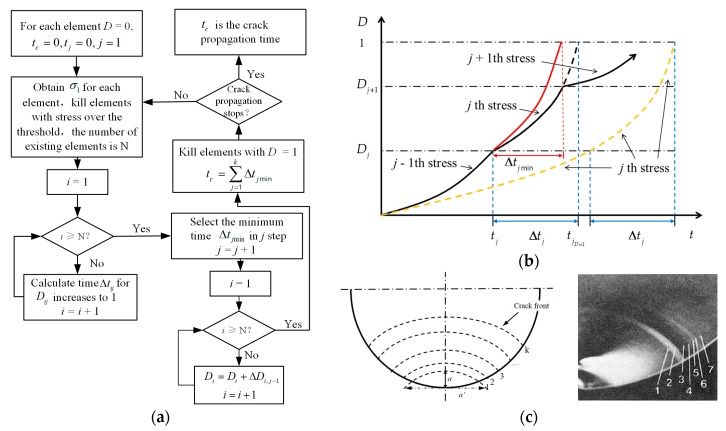
The calculation procedure for the crack propagation time and morphologies of the crack propagation states: (**a**) calculation procedure of the damage values and the crack propagation times; (**b**) computational procedure of the accumulated damage; (**c**) diagrammatic sketch and micrograph of the different crack propagation states.

Based on the definition and revolution law of the damage variable, the maximum principal stress of each element is directly obtained after one calculation cycle from the finite element model. The calculation procedure of the damage values and the crack propagation times is proposed in Figure 4a. The *t_c_* is the crack propagation time and $\Delta t_{ij}$ is the time for the damage to increase to one for element *i* at the *j*th step.

Moreover, the computational procedure of accumulated damage for *i*th element is shown in Figure 4b (*i* is omitted). The black solid curve represents the actual damage-increasing track under the changing stress, while the yellow dotted line is the virtual damage-increasing track under *j*th stress along with time. It can be observed from Figure 4b that the partial black solid line from *D_j_* to *D_j+1_* is parallel to part of the yellow dotted line. Since the damage development trend is only related to the current stress level and accumulated damage *D_j_*, the rate of damage change of the actual track from *D_j_* to *D_j_*_+1_ shown by the black solid line should be exactly the same as that of the virtual path shown by the yellow dotted line. This means that in the same time interval $\Delta t\;(\Delta t<\Delta t_{j\min})$, the damage calculation based on the black solid line is equal to that based on the yellow dotted line.

To calculate the increased damage from *D_j_* to *D_j_*_+1_ at the step *j*, the time-of-damage variable from *D_j_* to 1, noted as $\Delta t_{j}$, is calculated for each element respectively. According to Equation (6), $\Delta t_{j}$ can be easily calculated through the virtual path. Furthermore, the shortest time $\Delta t_{j\min}$ among all elements is selected as the time step for the cycle. The red dotted curve is the damage-increasing path of the element with $\Delta t_{j\min}$. For every element around the crack tip we can obtain the damage *D_j_*_+1_ after calculation cycle *j* through the yellow curve based on Equation (6). The total crack propagation time is the summary of the entire time steps. The fully damaged elements are killed in the finite element model and the crack profile is modified accordingly for the next cycle.

The calculating process iterates until the glass core is completely fractured, as in the process shown in Figure 4c. In the initial phase, there is a stress-concentration region on the glass core surface on account of the crack defect in the polymer coating. As damaged elements get killed along with time, the crack gradually extends deeper and wider. According to the work of Muraoka et al. [34], once the crack propagates to a certain length in the optical glass core, it becomes unstable and rapidly expands, resulting in fast fracture. Based on the observed micrographs of the fractured surface of an optical fiber by Muraoka and Abe [35], the boundary between the stable and unstable crack-propagation stages is 32 μm in the diagrammatic sketch shown in Figure 4c [36]. Therefore, the failure criterion is that crack depth reaches 32 μm for the glass core with 125 μm diameter.

## 3. Simulation Results and Discussion

### 3.1. Results at Different Bending Radii

**Figure 5:**
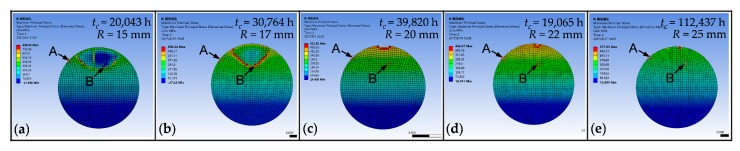
The fracture surface morphologies for different bending radii at 29,411 h and positions of selected elements. (**a**) *t_c_* = 20,043 h, *R* = 15 mm; (**b**) *t_c_* = 30,764 h, *R* = 17 mm; (**c**) *t_c_* = 39,820 h, *R* = 20 mm; (**d**) *t_c_* = 19,065 h, *R* = 22 mm; (**e**) *t_c_* = 11,2437 h, *R* = 25 mm.

In this section, under the condition of 2 N tensile force, the bending radii are set at 15 mm, 17 mm, 20 mm, 22 mm and 25 mm to compare the effect of bending radius on crack propagation time. The maximum principal stress contour plots at 29,411 h are illustrated in Figure 5. It can be seen that the glass core under *R* = 15 mm is already fractured; the crack under *R* = 17 mm is quite deep and presents an elliptical shape (see Figure 5b), which is probably due to stress relief of the cracked flank after it expanded. Meanwhile, there is only a tiny crack on the surface of the glass core when *R* = 20 mm and *R* = 22 mm, and no element is fully damaged under *R* = 25 mm.

**Figure 6:**
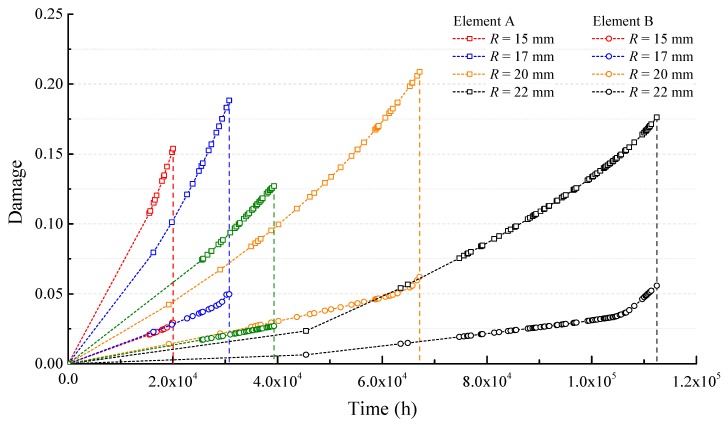
The damage of elements A and B vs time in the case of different bending radii *R*.

Since the killed elements cannot be shown in the diagram, it is hard to know the damage values at different locations around the fracture profile. Thus, element A and element B existing in different places shown in Figure 5 are selected to give examples of the time-varying damage around the crack profile, as illustrated in Figure 6.

Compared with the crack propagation times *t_c_* in Figure 5, there is an obvious inversely proportional relationship between the crack propagation time and the bending radius. This is because a smaller bending radius causes a higher bending load on the optical fiber, and it increases the damage-accumulating speed, as shown in Figure 6.

The accumulated damage values of elements A and B at the fracture moment are presented in Figure 6. It shows that element A acquires more severe damage than element B. The damage of element A is positively correlated to the crack propagation time and bending stress, while the bending stress is negatively correlated to the crack propagation time. Therefore, the final accumulated damage of element A with different bending radii cannot present an obvious monotone trend. For element B, the maximum damage merely reaches 0.055 for *R* = 25 mm, while the minimum is *D* = 0.026 when the bending radius is 20 mm. It seems that the difference of damage-growth rates is insignificant under different radii.

**Figure 7:**
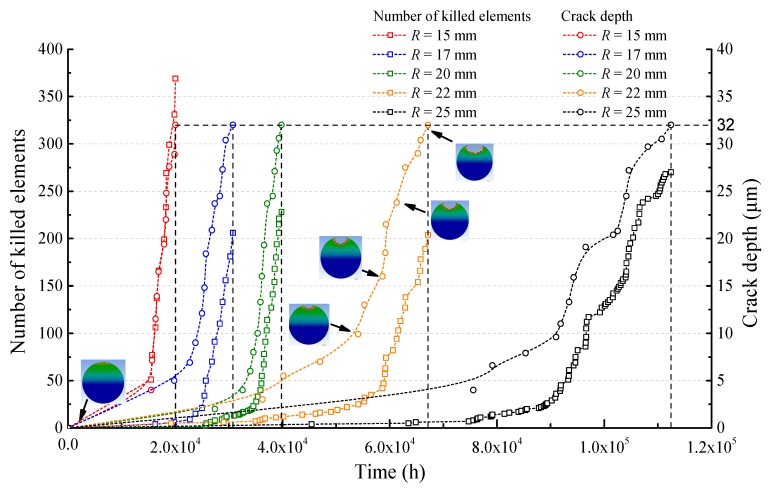
Crack depth and the number of killed elements vs time in the case of different bending radii *R*.

Moreover, the increasing crack depth as well as the number of elements killed with time are indicated in Figure 7. Failure times range from 2.00 × 10^4^ h to 1.12 × 10^5^ h, indicating that the bending radius has a major impact on the crack propagation time of the optical fiber. The average crack growth rates under *R* = 15 mm and *R* = 25 mm are 1.59 × 10^−^^3^ μm/h and 2.85 × 10^−^^4^ μm/h, respectively. Take the case of *R* = 22 mm, for example; the crack propagation process is presented with cracked cross sections at different time points. As the crack grows deeper and wider along with time, the local stress distribution is modified accordingly.

Obviously, two stages can be observed in the crack propagation process. Before about 30 elements are killed, the crack growth rates are relatively low, which corresponds to the crack-initiation stage. It takes a long time for the material to be damaged and the crack to grow. However, in the second stage, the crack growth rates increase rapidly as elements are killed faster, and this can be described as the early crack-extension stage. In the crack-initiation stage, the crack depth prediction is reliant on the element size, such that a smaller element size could provide a more accurate prediction.

### 3.2. Results at Different Tensile Forces

**Figure 8:**
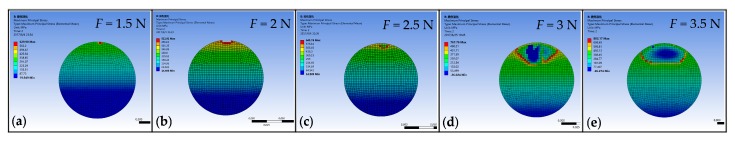
The maximum principal stress maps and crack shapes for five loads (*F* = 1.5, 2, 2.5, 3, 3.5 N) at 18,999 h. (**a**) *F* = 1.5 N; (**b**) *F* = 2 N; (**c**) *F* = 2.5 N; (**d**) *F* = 3 N; (**e**) *F* = 3.5 N.

**Figure 9:**
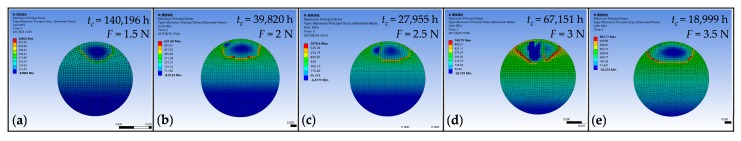
The maximum principal stress maps and crack shapes for five loads (*F* = 1.5, 2, 2.5, 3, 3.5 N) at the failure times. (**a**) *t_c_* = 140,196 h, *F* = 1.5 N; (**b**) *t_c_* = 39,820 h, *F* = 2 N; (**c**) *t_c_* = 27,955 h, *F* = 2.5 N; (**d**) *t_c_* = 67,151 h, *F* = 3 N; (**e**) *t_c_* = 18,999 h, *F* = 3.5 N.

Under the condition of bending radius *R* = 20 mm, five external tensile forces are applied to the optical fiber for analyzing the effect of external tension on life prediction. One application of optical fibers, the fiber optic hydrophone, should acquire a coiled tensile force above 0.9 N to guarantee that the optical fiber is fixed in place even under an external disturbance. Herein, we assume that the external coiled forces *F* respectively are 1.5 N, 2 N, 2.5 N, 3 N and 3.5 N. The crack-propagation states and maximum principal stress maps for the five loads at the time *t* = 18,999 h are displayed in Figure 8. While the glass cores under 3 N and 3.5 N tensile force are almost ruptured, there are merely a few elements that are fully damaged and the crack length is quite small under 2 N and 2.5 N tensile loads. Figure 9 exhibits the maximum principal stress distribution and crack shapes for five loads at the crack propagation time. It shows that a higher tensile force results in a shorter crack propagation time.

By comparison among the fractured states and crack propagation times shown in Figure 9, an inversely proportional relationship can be found. Higher tensile forces causes higher stress levels around the defect, which further gives rise to higher element killing rates and damage-accumulating speed. Therefore, higher *F* results in a shorter crack propagation time.

Despite the effect of element size, cracks propagate to the flanks as they get deeper, and the cracked cross sections in Figure 9 approximately appear as a penny shape, which conforms to the actual micrograph of the fractured surface of an optical fiber observed by Muraoka and Abe [36], as shown in Figure 9. Additionally, a higher tensile load induces a bigger cracked area.

**Figure 10:**
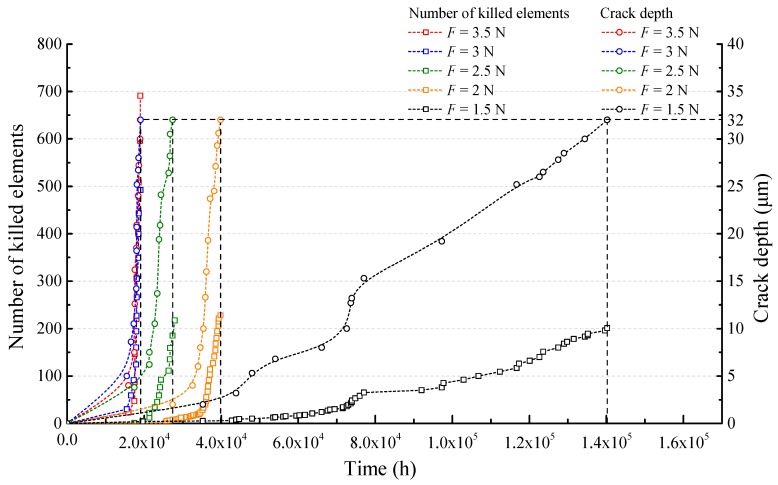
Crack depth and number of elements killed vs time in the case of different loads *F*.

Figure 10 illustrates that the crack depth and the number of accumulated elements killed increases over time. The early stage of the crack propagation process takes up a large proportion of the optical fiber’s total crack propagation time. As damage values increase to a relatively high level, the time step for one iteration gets shorter and more elements are killed in a limited time interval, representing a higher crack-growth speed.

There is a notable growth of the number of killed elements in the finite element models under an increasing load. Only 201 elements are killed for *F* = 1.5 N, while over 600 elements are fully damaged until fracture for *F* = 3.5 N. For the optical fiber subjected to a larger tensile load, both elements at the surface and center of the glass core obtain faster damage accumulation under a higher stress level, which results in more elements being killed.

### 3.3. Results at Different Optical Fiber Diameters

Optical fibers with multiple diameters are manufactured for various objectives. Some scholars investigated the experimental results and concluded that thinner optical fibers have smaller bending loss, while thicker ones can possess higher transmission capacity. Few researchers focus on the relationship between the geometry size of the optical fibers and their usage time. Thus, in this section, five commercial optical fibers with different geometry parameters are considered to discuss the geometry size effect on the optical fibers’ crack propagation times through the finite element model. The geometry parameters and the critical crack depths of these optical fibers with different diameters are respectively listed in Table 1. The critical crack depth listed in Table 1 represents the critical crack depth of the turning point beyond which the crack propagation behavior transits from the stable region to the unstable region. In general, the crack grows fast and unstably at the last region and leads to sudden failure. Therefore, the time duration of the unstable region is much shorter compared with that of the stable crack propagation region, and can be ignored in most of studies [34,35].

**Figure 11:**
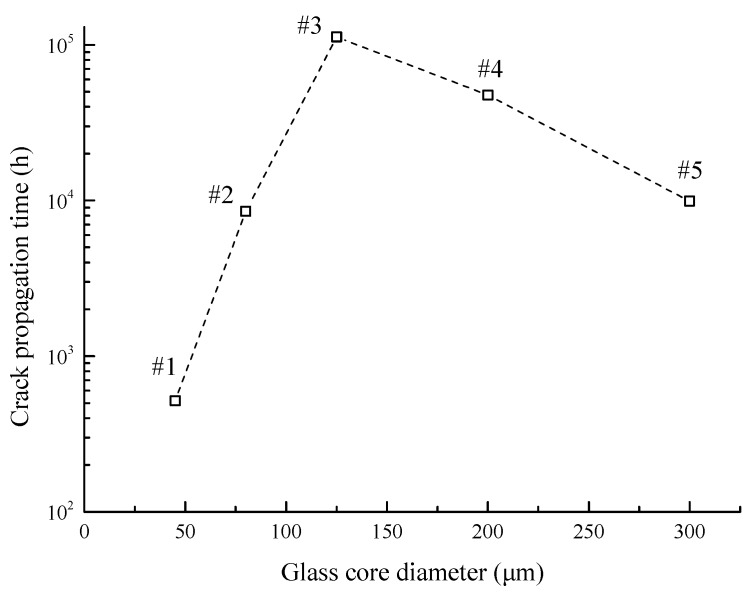
Crack propagation time vs. glass core diameter.

All the simulations are carried out under the condition that *F* = 2 N and *R* = 25 mm. The relationship between the crack propagation time and glass core diameter is shown in Figure 11. From the simulation results, we can see that in the initial part of the curve, with the diameter of the optical fiber core and cladding diagram increasing, the crack propagation time does not show a correlational variation trend.

**Figure 12:**
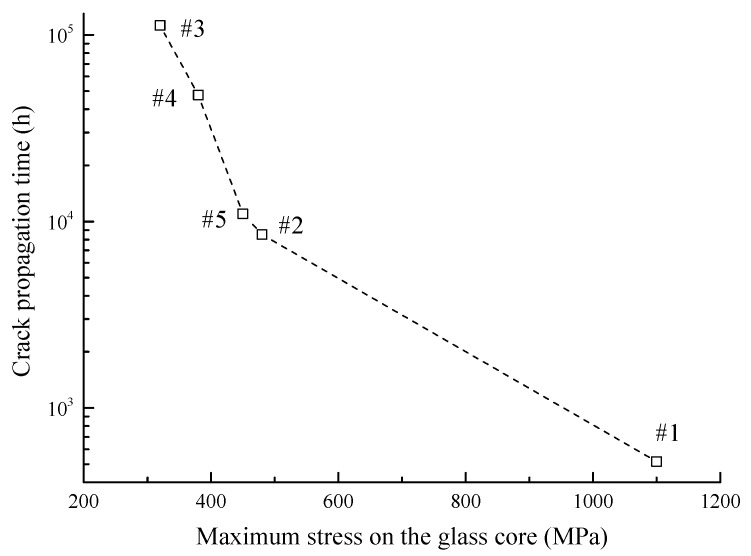
Crack propagation time vs. maximum stress on the glass core.

**Figure 13:**
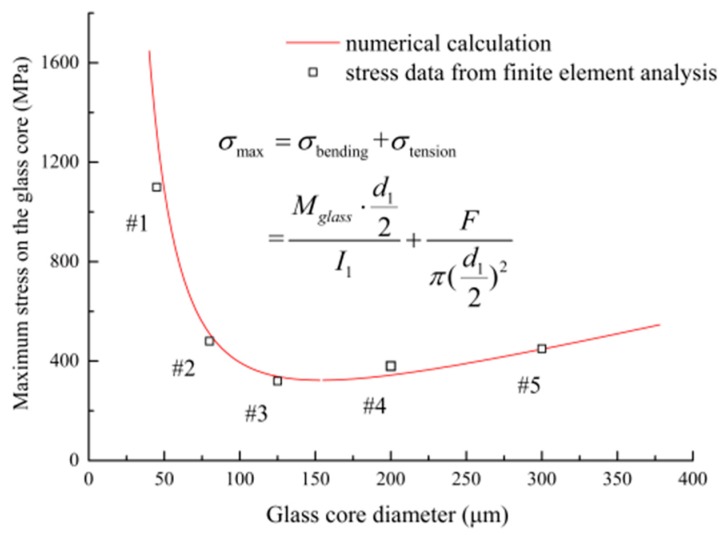
Maximum stress on the glass core vs. glass core diameter.

For deeply investigating the variety of propagation times, we are trying to compare the stress levels of glass cores with different diameters. The simulation results in section III-A and III-B prove that the stress on the glass core has a significant impact on the crack propagation time of the optical fiber. As shown in Figure 12, there is a negative relationship between the crack propagation time and the maximum stress on the glass core (obtained by the finite element analysis). A higher stress level leads to a greater damage-accumulating speed that causes more elements to be killed, which results in a shorter crack propagation time. In addition, based on the finite element results, the relationship between the diameter and the maximum stress level, as shown in Figure 13, can also explain the varying trends of propagation time in Figure 11. For instance, having the minimum stress level in these five cases, the #3 optical fiber has the longest propagation time.

In actuality, the maximum stress on the glass core is determined by the combination of external loads, optical fiber geometry parameters and defect size. It approximately equals the sum of the maximum bending stress and the tensile stress on the glass core, which can be deduced as the function of glass core diameter *d*_1_, as shown in the equation of Figure 13. The *I*_1_ is the inertia moment of the glass core. From Figure 13, the finite element results are well matched with the numerical calculation. It can be seen that the maximum stress on the glass core decreases first and then increases along with the glass core diameter. The lowest point of the curve corresponds to the diameter of 154 μm, which is close to the diameter of the #3 optical fiber.

In summary, there is an optimum diameter for the glass core that acquires the lowest stress, and lower stress levels would give longer crack propagation times. On the basis of these two facts, the optical fiber with a 125 μm-diameter glass core has the longest crack propagation time. This conclusion could give optimization suggestions in optical fiber design to improve its lifetime under the static stress condition.

## 4. Experimental Verification

### 4.1. Experimental Procedure

**Figure 14:**
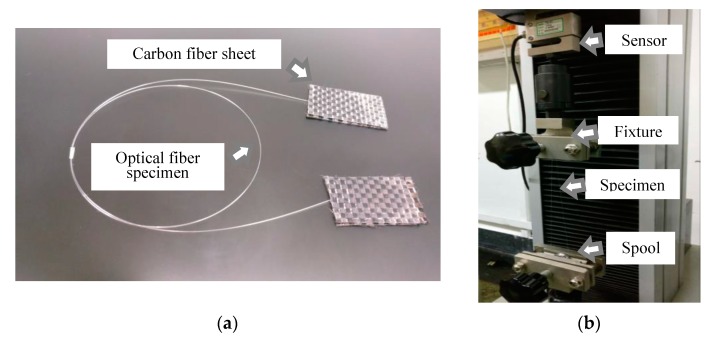
The test specimen and experimental devices: (**a**) optical fiber specimen and carbon fiber sheets; (**b**) experimental devices and the way to apply loads.

A glass core with diameter *d*_1_ = 125 μm is used to verify the simulation results in section III-A and III-B. As shown in Figure 14a, both sides of the samples are glued to carbon fiber sheets with epoxy resin to protect them from the crush caused by fixtures. The samples are subjected to both bending and tensile loads the same as the simulation conditions in a laboratory environment (25 °C and 40% relative humidity).

Figure 14b exhibits the experiment device and the way to apply loads. Both carbon fiber sheets are clamped by fixtures while the optical fiber wound on the spool is fastened tightly by the other pair of fixtures. A preset crack, such as the one in Figure 3, is introduced by thin blades. The geometry parameters of the preset crack are inspected using a stereoscopic microscope. The average crack width 2*l* (see Figure 3a) is around 0.146 mm, and the opening angle θ of the crack defect (see Figure 3b) is smaller than 3°. The average depth of the crack defect is calculated based on the geometry of the blade, which is around 0.059 mm. Since the simulation results indicate that the crack propagation times are over 10,000 h, to reduce the experimental time and cost, higher tensile loads are applied in these cases.

In the first case, the bending radii *R* are set to 15, 17, 20, 22 and 25 mm and the tensile load *F* is 15 N. In the second case, the bending radius *R* is 20 mm and the tensile loads are selected from 9 to 15 N. The testing process is continually monitored by the computer to keep the loads stable at the preset values until the optical fibers fracture.

The fractured cross sections are then imaged with a Hitachi S-4800 scanning electron microscope (SEM). The SEM images provide the information of crack geometry and initiations of the fiber fracture.

### 4.2. Results and Discussion

**Figure 15:**
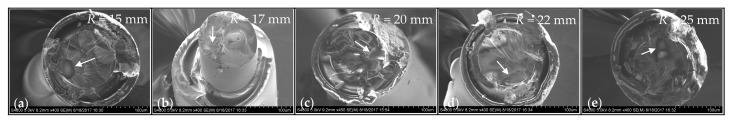
Fracture cross sections under different bending radii. (**a**) *R* = 15 mm; (**b**) *R* = 17 mm; (**c**) *R* = 20 mm; (**d**) *R* = 22 mm; (**e**) *R* = 25 mm.

SEM images of fractured cross sections under different bending radii are illustrated in Figure 15. As shown in the images, there is no visible boundary between the core and cladding. The core is covered tightly by two polymer coatings.

Cracks initiate from the preset defects in the coating and gradually propagate until they cause a complete fracture. There are two phases of the fracture process, which can be observed in the SEM images. The first stable propagation phase results in a smooth penny-shaped crack, and the second unstable crack extension phase is reflected on the radial cracks on the fractured cross section. The boundaries of the two phases are pointed out with arrows. It can be seen that the average depth of cracks is 32 μm with an acceptable disparity. Despite the normal fracture features, peeling of the coating layer can be observed while *R* = 17 mm. This is mainly due to the weak bonding strength between the coating and the glass core, and its effect should be further studied.

**Figure 16:**
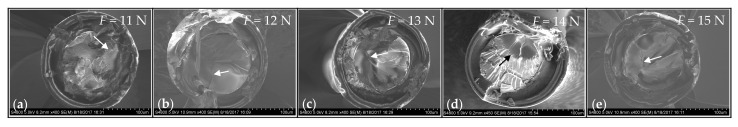
Fracture cross sections under different tensile forces. (**a**) *F* = 11 N; (**b**) *F* = 12 N; (**c**) *F* = 13 N; (**d**) *F* = 14 N; (**e**) *F* = 15 N.

Specimens tested under different tensile forces are also observed with SEM examinations, and the fracture morphologies are shown in Figure 16. As the tensile force increases, the cracks are getting wider, caused by the higher stress on the side of the crack, which basically validates the simulation results shown in Figure 9. Based on the simulation results, greater tensile forces can easily cause elements on the crack flank to take on higher damage values and be killed, which leads to the appearance of wider cracks.

**Figure 17:**
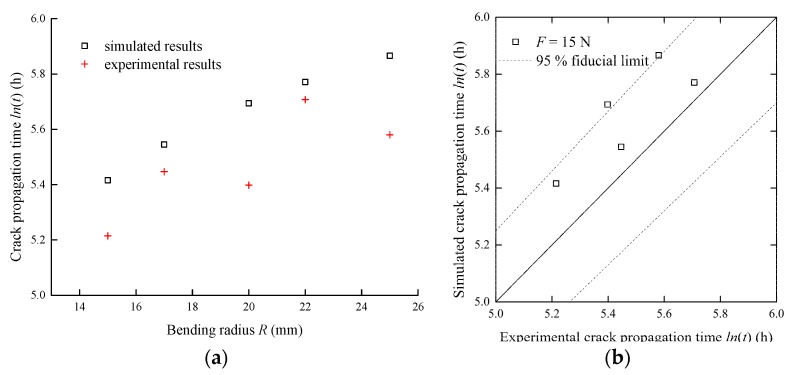
The comparison between the simulated and experimental crack propagation times under different bending radii: (**a**) crack propagation time vs bending radius; (**b**) simulated results vs experimental results.

To verify the proposed method, the crack propagation time versus bending radius for both the simulation and experiment are presented in Figure 17a. The increasing trend of crack propagation time along with the bending radius can be observed from simulated and experimental data. To further quantify the simulation accuracy, results from both the simulation and experiment are shown in Figure 17b. The central diagonal solid line represents an ideal agreement between the simulated and the experimental results. The dashed lines denote the 95% fiducial limits. It can be seen that most points are within the fiducial limits. Moreover, it should be noticed that the experimental crack propagation times are shorter than the simulated results. This phenomenon may be caused by uncertainties associated with the simulation and experiment. Uncertainties arise from a variety of sources; they can affect the experimental result, which differs from the simulated crack propagation due to, for example, variability in the preset crack procedure. The measured data can also be affected due to the fluctuations of the surrounding environment.

**Figure 18:**
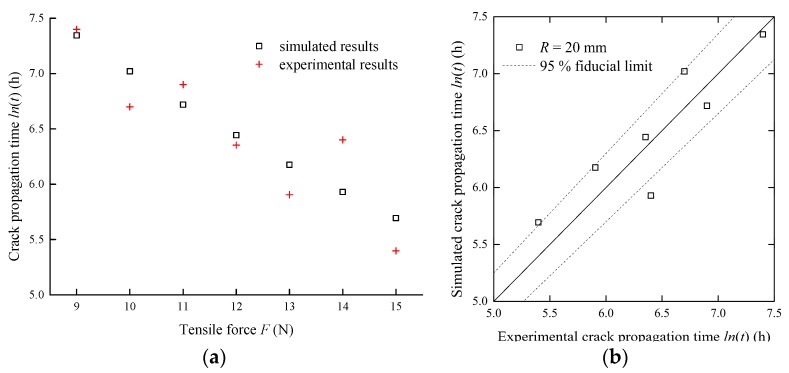
The comparison between the simulated and experimental crack propagation times under different tensile forces: (**a**) crack propagation time vs tensile force; (**b**) simulated results vs experimental results.

In addition, Figure 18a shows the evolution of crack propagation time under various tensile forces. The crack propagation times decrease with the increasing tensile forces. The simulated crack propagation times are close to the experimental values. Some discrepancy may also exist for the above mentioned reasons. Moreover, simulated results versus experimental results under various tensile forces are presented in Figure 18b to quantify the simulation accuracy. Most points are within the 95% fiducial limits. Therefore, it is convincing that the proposed approach can quantitatively predict the crack propagation times of the optical fibers under the experimental conditions.

Therefore, for the twining of optical fiber in a hydrophone, lower tensile force and greater bending radius would increase the crack propagation time of optical fiber remarkably. For the circumstance where bended optical fibers are composed as the key part of the sensor, longer lifetime could be obtained by improving the structure to decrease the stress on the glass core.

## 5. Conclusions

Optical fibers are widely investigated and applied in both telecommunication and sensor industries due to their prominent properties. For satisfying the long-term usage requirement of optical fibers under critical operating conditions, it is essential to make an accurate failure-time prediction for optical fibers in sensors. In this paper, an approach based on continuum damage mechanics is presented for calculating the crack propagation process and failure time of the optical fiber under static bending and tensile loads. The finite element model of the optical fiber subsection is built with a pre-existing crack defect introduced in post-processing. The maximum principal stress is used to calculate the damage variables for each element based on the time-varying damage revolution mechanism. The crack propagates by killing damaged elements in the finite element model until the fracture threshold is reached. Finally, the test data validate the results from the three-dimensional simulation under different bending and tensile loads well. It is believed that the proposed method could give an intuitionistic process of optical fiber crack propagation and provide effective improvement countermeasures for long-term usage of optical fibers. The main conclusions are summarized below:

(1) From the simulation results, the change of the crack-front shape in the propagation process is consistent with the observed image in the actual test, which indicates that the traces of the crack fronts present a fan-like pattern path.
(2) The crack propagation process can be divided into two stages. Firstly, the crack-initiation stage lasts from the starting point to the moment that nearly 30 elements are killed, and the crack growth rates are relatively low, since a long time is taken for the material to be damaged in the first place. Then, once entering the early crack-extension stage, the crack growth rates increase rapidly as elements are killed faster until the failure criteria are met.
(3) Under different bending radii, the simulated crack propagation time values show that there is an obvious inversely proportional relationship between the crack propagation time and bending radius. The reason is that a smaller bending radius causes a higher bending load on the optical fiber, and it increases the damage-accumulating speed. Additionally, compared with the test data, the simulation results have the same variation tendency and similar magnitude changes in the range of acceptable errors.
(4) Based on the simulation results, greater tensile forces can easily cause elements on the crack flank to take on higher damage values and be killed, leading to the appearance of wider cracks. The phenomenon basically conforms to the test results. In addition, the simulated crack propagation times are close to the extrapolated values from fitting the curve of the experimental results.
(5) The geometry size of optical fibers manufactured can seriously affect the optical fibers’ crack propagation times through the finite element analysis. Through deeply investigating simulation results, the maximum stress on the glass core decreases first and then increases along with the diameter of the glass core, which can explain the non-linear relationship between the crack propagation time and glass core diameter. Thus, there is an optimum diameter for the glass core that takes on the lowest stress, which could give an optimization policy in the size design of optical fibers to improve their usage time under static loads.

## Figures and Tables

**Table 1:** Geometry parameters of the five optical fibers and critical crack depths.

	#1	#2	#3	#4	#5
Glass core diameter (μm)	45	80	125	200	300
Inner coating diameter (μm)	77	120	187	230	330
Outer coating diameter (μm)	115	165	245	500	650
Critical crack depth (μm)	11	22	32	50	79
